# Communicable diseases intelligence.

**DOI:** 10.3201/eid0101.950109

**Published:** 1995

**Authors:** H Longbottom


### Communicable Diseases Intelligence

Communicable Diseases Intelligence (CDI) is a fortnightly publication of the Australian Department of Human Services and Health and the Communicable Diseases Network of Australia and New Zealand. The Network comprises representatives of the Australian Department of Human Services and Health, the State and Territory health authorities, and other organizations involved in communicable disease surveillance and control from throughout the country. In addition, there is a representative from New Zealand. It has fortnightly teleconferences and other meetings to exchange information on emerging communicable disease activity and to coordinate surveillance and control activities.

Each issue of CDI incorporates reports from Australia's national communicable diseases surveillance systems, including the National Notifiable Diseases Surveillance System, the CDI Laboratory Reporting Schemes, and the Australian Sentinel General Practitioner Surveillance Network. Reports from the National Salmonella Surveillance Scheme, the Australian Gonococcal Surveillance Programme and the National HIV, AIDS, and Tuberculosis Reporting Systems are also regularly included.

CDI also publishes timely reports of communicable disease outbreaks and other articles dealing with a wide range of subjects relevant to the surveillance and control of communicable diseases in Australia. Recently published items have reported, for example, the first identification of endemically acquired hepatitis E in the Northern Territory of Australia, an outbreak of influenza in a nursing home, the epidemiology of hepatitis A in South Australia, the epidemiology of Barmah Forest virus disease in Western Australia, and the outbreak of respiratory disease in humans and horses due to a previously unrecognized paramyxovirus.

CDI is available from
The Editor
Communicable Diseases Intelligence
AIDS and Communicable Diseases Branch
Department of Human Services and Health
GPO Box 9848
Canberra ACT 2601
Australia

**Helen Longbottom**
Department of Human Services and Health
Canberra ACT Australia

### DxMONITOR: Compiling Veterinary Diagnostic Laboratory Results

The DxMONITOR is a collaborative effort between the U.S. Department of Agriculture, Animal and Plant Health Inspection Service's Veterinary Services (USDA:APHIS:VS), the American Association of Veterinary Laboratory Diagnosticians, and the United States Animal Health Association. This quarterly animal health report presents compiled data from national animal disease control and eradication programs (bovine and porcine brucellosis, bovine tuberculosis, porcine pseudorabies and equine infectious anemia); patterns of selected diseases based on veterinary diagnostic laboratory data (bovine leukosis; bovine bluetongue; bovine, ovine and caprine paratuberculosis; equine arboviral encephalitis; equine viral arteritis; porcine reproductive and respiratory syndrome); data on selected etiologic agents associated with specific animal health events such as bovine abortion; global disease distribution (bovine spongiform encephalopathy); and notes from veterinary diagnostic laboratories about unusual laboratory findings or new diagnostic procedures.

The DxMONITOR has contributed to a greater awareness of animal diseases in the United States. Global trade agreements, the worldwide information explosion, and increasing public concern over the safety and quality of food have focused attention on animal health. Compilation of veterinary diagnostic laboratory data is one component of the USDA: APHIS efforts to respond to these increased demands for animal health information through an integrated and coordinated monitoring and surveillance system. Animal-health monitoring and disease surveillance concern not only animal health per se, but also interactions with the environment, animal welfare, production practices and product wholesomeness which impact animal health. The DxMONITOR is mailed to all interested parties without charge and is increasingly available through electronic dissemination channels. For more information or subscription, contact DxMonitor Animal Health Report, c/o Centers for Epidemiology and Animal Health, USDA:APHIS:VS, 555 S. Howes, Suite 200, Ft. Collins, CO 80521-2586; telephone 303-490-7800; e-mail DXMONITOR@aphis.ag.gov.

**William Hueston**
Centers for Epidemiology and Animal Health
Veterinary Services
APHIS, U.S. Department of Agriculture
Fort Collins, Colorado, USA

